# Differences in labour participation between people living with HIV and the general population: Results from Spain along the business cycle

**DOI:** 10.1371/journal.pone.0195735

**Published:** 2018-04-23

**Authors:** Luz María Peña Longobardo, Juan Oliva-Moreno

**Affiliations:** Department of Economic Analysis and Finance, Faculty of Law and Social Sciences, University of Castilla-La Mancha, Toledo, Spain; Public Health Agency of Canada, CANADA

## Abstract

**Background:**

HIV/AIDS (Human immunodeficiency virus/Acquired immune deficiency syndrome) not only has a strong impact on the health of the worldwide population but also affects the labour status of HIV-positive people. The primary aim of this paper is to compare the labour participation of people living with HIV (PlwHIV) with the labour participation of the general population along the last business cycle in Spain.

**Method:**

The data used are from the Hospital Survey on HIV-AIDS, with a total sample size of 4,651 PlwHIV and the Labour Force Survey from 2001 to 2010, with a total sample size of 660,674 individuals as general population. Propensity Score Matching method was used to analyse the differences between the labour participation of PlwHIV and the general population. Additionally, several specific models categorised into different subgroups (gender, education, source of infection and level of defences) were also performed.

**Results:**

We identified a convergence in labour participation across the period in the two populations considered: PlwHIV was 23% less likely to have a job than the general population during 2001–2002 and 14% less likely during 2009–2010. This convergence is mainly explained by two facts: first, the positive evolution of people infected by sex; second, the change in the PlwHIV population composition with a decreasing weight of people infected by drug use throughout the decade. Thereby, at the end of period, there was no statistical difference in the employment rate between PlwHIV infected through sex and the general population but there was strongly difference in PlwHIV infected through drugs.

**Conclusion:**

Inmunological status, source of infection and level of education play a relevant role among the PlwHIV population when comparing their labour participation with the general population. In spite of this positive result, the likelihood of being employed in HIV-positive people continues to be different from that of non-carriers. Our study shows that institutional features of labour markets are relevant and should be considered in comparison between countries.

## Introduction

Labour participation has been identified as a significant social issue facing people living with HIV (PlwHIV). A notable characteristic of this population group is that the vast majority are of working age. More specifically, it was estimated that almost 90% of PlwHIV worldwide are between 25 and 49 years old [1].

Until the introduction of highly active antiretroviral treatments, disease progression would have driven out a vast majority of PlwHIV from the labour force [2–5]. Therapeutic advances since the 1990s have substantially increased life expectancy and improved the living standards of this population. Consequently, the labour participation of PlwHIV has clearly improved, increasing the likelihood of keeping a job and reducing the difference in the employment rate among PlwHIV and the general population [6–10].

Nevertheless, although therapeutic advances have gradually improved this population group’s labour integration, this does not mean that being diagnosed with HIV has no effect on labour participation. Several studies have suggested that the decline in the percentage of PlwHIV employed may be a rational consequence of adapting their expectations to a negative health shock that implies a shorter life expectancy [11]. Other papers have highlighted the association between health status and likelihood of employment. The immunological status but also the psychological aspects [12–18], as well as the importance of other diseases, physical problems or the presence of fragility associated with premature ageing are relevant aspects to explain the probability of employment [17, 19–21]. In addition to these findings, the literature agrees that in addition to health status, other socioeconomic characteristics, such as age, sex or level of education, are as important as the previous ones to explain the labour participation of PlwHIV [9, 10, 12, 14].

The positive progression of PlwHIV labour participation over the last decade could be affected by changes in business cycle. As literature has showed, the recent economic crisis had played a relevant role in health and labour markets for non-HIV people, especially for those with poor health status or with chronic diseases [22–27]. Moreover, a recent study has concluded that improvements in HIV care are not necessarily conducive to significant improvements related to the employment status of PlwHIV [28]. This finding could be due to the recent economic crisis. Despite advances in medical treatment, at a time of economic recession, the unemployment rate of PlwHIV in France has been affected by a greater extent than that of the general population [29]. The authors conclude that in time of economic crisis, a part of PlwHIV could have been excluded from the labour market.

It is important to stress that economic crisis in Europe has been asymmetric. The duration and depth of the crisis has been different across countries and their labour markets have behaved very differently. For example, in Spain, the economic crisis began in 2008 and, according to figures from the National Statistical Institute, concluded in 2014. By the end of 2016, the Spanish economy had not recovered GDP values prior to the crisis [30]. The Spanish labour market has been particularly affected by the crisis, as the unemployment rate has sharply increased (from 8% in 2007 to 25% in 2014), while employment has decreased from 2007 to 2014 [31].

The objective of our study is to compare the labour participation of PlwHIV residing in Spain with the labour participation of the general population along the business cycle. In this way, we want to test whether the result found by Annequin et al in France [29] is comparable with the Spanish crisis or if the asymmetry in the economic crisis and labour markets across countries leads to differential results. Thus, we will investigate whether the differences in employment rate between PlwHIV vs the general population have been increased or decreased over the last decade, comparing three different points of time: before the economic crisis (2001–2002 and 2006–2007) and during the first years of the economic crisis (2009–2010) in Spain. Conversely, another objective of our work is to identify profiles for which the differences in the employment rate between PlwHIV and the general population have decreased over the period studied and, on the contrary, profiles where the differences in labour have become more acute.

## Methods

### Data

Two different datasets were used to carry out this analysis, one to obtain information related to PlwHIV and another to acquire information regarding the general population. For the first group, the Hospital Survey on HIV/AIDS patients by the General Secretariat of the AIDS Plan [32] was used. The aim of this survey was to analyse the socio-demographic characteristics of PlwHIV. The survey was a one-day cross-sectional study (repeated on an annual basis) where information was collected using a questionnaire completed by the physician during or immediately after the patient’s consultation. Between 69 and 86 centres participated in this survey during the period under analysis with a total sample size of 4,651 PlwHIV. The target population was people with HIV/AIDS, diagnosed and treated in hospital outpatient departments, day hospital centres or hospital admissions. PlwHIV receiving hospital treatment for reasons unrelated to HIV were not included. The information collected by the Hospital Survey on HIV/AIDS contains clinical information on immunological status, such as the level of defences measured by CD4 cell count (CD4 count/μl≥500 indicates high level of defences, 200≤CD4/μl <500 indicates medium level of defences and 200≤CD4/μl indicates low level of defences) and most likely cause of transmission. There is also information related to demographic variables such as gender, age, educational level and employment status.

To acquire information about the employment status of the general population, the Labour Force Survey from 2001 to 2010, as performed by the Spanish Statistics Institute [33], was used. This survey was designed to provide detailed results on a national level. The total sample for the period under analysis was 660,674 individuals for the years selected. Performed every three months, the study considered the population living in family dwellings on Spanish national territory. The study’s main aim is to reveal the characteristics of that population group with regard to the labour market, collecting information, such as gender, age, marital status, education and other individual characteristics. Although the Labour Force Survey does not provide information on whether or not respondents are HIV-positive, since the prevalence rate of HIV among the general Spanish population was 0.3% for this period, we can assume a margin of error that the respondents are not HIV-positive [34].

Because the analysis is focused on labour participation, we only included individuals in their working age (16–64 years old) and PlwHIV who do not live in prison (that population reached just 3% of the total PlwHIV).

### Variables

The main variables used in the analysis are; a dichotomous variable indicates whether individual works or not, gender, age (from 16 to 64 years old), level of education (no education, primary, secondary and tertiary education), and whether an individual is in treatment group or control group (1 if the individual suffers from HIV, 0 otherwise).

### Statistical analysis

Propensity Score Matching method was used to analyse the differences in the labour participation of PlwHIV residing in Spain compared with the labour participation of the general population during three different periods of time: years 2001–2002, 2006–2007 and 2009–2010, using logistic regressions to estimate the propensity scores. Due to the treatment assignment (HIV-positive) being not a random process and the differences between individuals in the treatment and control groups were recorded with observable variables (such as age, gender or education), a matching method was therefore the most suitable method [35–37]. The idea was that individuals from the control group (general population) were matched to individuals in the treatment group (those with HIV); thus, the key assumption was that for each treated individual, an untreated individual could be found.

Since we observed either treated (HIV-positive) or untreated (general population) individuals, we were unable to note the causal effect of interest, Y_i1_-Y_i0_. We may thus consider the Average Treatment Effect on the Treatment Group (ATE): *E*(*Y*_1_ − *Y*_0_|*X*,*D* = 1) = *E*(Δ|*X*,*D* = 1), where D is a dummy variable indicating status as HIV-positive (1) or part of the general population (0). This magnitude measures the difference in the average outcome (being employed) of those HIV-positive individuals and those individuals from the general population.

Propensity Score Matching method may have been difficult to accomplish if determined by multiple variables (meaning matching all participants in the treatment group with all participants in the control group with the same characteristics). To avoid this problem caused by dimensionality, it was necessary to use the propensity score for determination purposes, demonstrating that if (*Y*_0_, *Y*_1_) ⊥ *D*|*X* and 0 <P (X) <1, where *P*(*X*) = Pr(*D* = 1|*X*), then (*Y*_0_, *Y*_1_) ⊥ *D*|*P*(*X*), i.e., the result regarding employment status is the same for individuals both with and without HIV once controlled for the X variables or for the propensity score, P (X) [38].

In brief, this technique compared in mean terms the employment rate between different groups considered in this analysis, controlling for socio-demographic characteristics (such as gender, age and level of education). What is more, it compares a woman with low level of education, 30 years old (for instance) and suffering from HIV with a woman with low level of education, 30 years old and not suffering from HIV. By this, it does compare similar individuals (with the same gender, age and level of education) with the only difference of suffering from HIV or not.

Having checked which matching estimator was the most suitable for carrying out our analysis (nearest-neighbour matching or Kernel matching), we opted for the nearest-neighbour matching technique, as it had lower pseudo-R squared of probit estimation for propensity score after matching and a lower number of treated individuals lost after matching [39]. The model used to estimate the propensity scores was logistic regressions. Additionally, it was used greedy nearest neighbour matching without replacement within specified caliper widths algorithms. Additionally, as some previous studies have shown [40] that algorithms that employ greedy neighbor matching within a caliper width may produce less biased estimates of effect when compared with other matching algorithms [40]. Therefore, it is highly recommended in the literature. Basically, this matches an individual in treatment group and an individual in control group only if the absolute difference in their propensity scores is within a specific maximal distance (caliper), which is defined as a proportion of the standard deviation of the logit regression of the propensity score [41, 42].

The software used to carry out the analysis was STATA 14. The software code used for the analysis is available under request.

## Results

### Sample characteristics

Table 1 shows the sample characteristics for PlwHIV and the general population for the three different periods analysed. During the period 2001–2002, approximately 46% of PlwHIV had a job (55% for general population). Similarly, during 2006–2007, the employment rate for PlwHIV was 51% (62% for general population), while during the first year of the economic crisis, the employment rate decreased until reaching 47% for PlwHIV (58% for general population). Regarding educational level, almost 60% had received either no education or education only to the primary level (30% in the case of the general population), whereas just 10% had received a tertiary education (21% for the general population). Within PlwHIV, the main cause of infection was through drug use followed by sex. Moreover, almost 30% had low level of defences. Table 1 also shows these characteristics during the business cycle.

**Table 1:** Sample characteristics for PlwHIV and the general population.

	Period 2001–2002		Period 2006–2007		Period 2009–2010	
	PlwHIV (percentage or average) n = 1,866	General population (percentage or average) n = 229,632	PlwHIV (percentage or average) n = 1,432	General population (percentage or average) n = 209,581	PlwHIV (percentage or average) n = 1,353	General population (percentage or average) n = 221,461
Employed	832 (45.99)	126,921 (55.27)	715 (50.93)	130,442 (62.24)	626 (46.96)	129,496 (58.47)
Age	1,866 (35.93)	229,632 (36.65)	1,432 (39.14)	209,581 (37.96)	1,353 (40.58)	221,461 (38.60)
Female	484 (26.76)	115,901 (50.47)	385 (28.35)	106,288 (50.71)	355 (27.37)	112,180 (50.65)
No education	39 (2.09)	3,504 (1.53)	45 (4.33)	1,866 (0.99)	37 (2.73)	1,994 (0.90)
Primary education	1,051 (58.00)	68,239 (29.72)	837 (56.47)	43,230 (20.78)	727 (54.73)	42,024 (18.98)
Secondary education	545 (29.21)	109,547 (47.71)	422 (29.47)	109,815 (52.62)	431 (32.86)	118,330 (53.43)
Higher education	181 (10.70)	48,342 (21.04)	125 (9.73)	53,039 (25.51)	131 (9.68)	59,113 (26.69)
Infected through sex	707 (37.88)	--	609 (42.52)	--	700 (51.73)	--
Infected through drug use	1,076 (57.68)	--	770 (53.77)	--	584 (43.18)	--
Infected through blood transfusion	20 (1.07)	--	11 (0.76)	--	8 (0.59)	--
Infected through unknown cause	63 (3.37)	--	42 (2.95)	--	61 (4.5)	--
Low level of defences	541 (30.19)	--	409 (28.56)	--	294 (21.73	--
Medium level of defences	702 (39.17)	--	556 (38.83)	--	500 (36.95)	--
High level of defences	549 (30.64)	--	467 (32.61)	--	559 (41.32)	--

Source: Designed using data from the Hospital Survey on HIV/AIDS and the Labour Force Survey.

### Statistical analysis

Table 2 shows non conditional employment rates between PlwHIV and general population for different profiles.

**Table 2:** Comparison of labour participation between PlwHIV and general population without controlling for socio-demographic variables.

	Period 2001–2002				Period 2006–2007				Period 2009–2010			
Sub-group	PlwHIV^1^	GP^2^	Diff^3^	95% CI	PlwHIV^1^	GP^2^	Diff^3^	95% CI	PlwHIV^1^	GP^2^	Diff^3^	95% CI
PlwHIV vs. general population	0.459	0.552	0.092	[0.069–0.115]*	0.509	0.622	0.113	[0.087–0.138]*	0.469	0.584	0.115	[0.088–0.1416]*
PlwHIV female vs. general female population	0.395	0.403	0.008	[-0.036–0.052]	0.399	0.506	0.107	[0.056–0.157]*	0.513	0.397	0.115	[0.063–0.167]*
PlwHIV male vs. general male population	0.486	0.704	0.217	[0.192–0.242]*	0.548	0.741	0.192	[0.164–0.220]*	0.492	0.658	0.165	[0.134–0.196]*
PlwHIV with low defences vs. general population	0.347	0.552	0.205	[0.162–0.248]*	0.350	0.622	0.272	[0.224–0.32]*	0.340	0.584	0.244	[0.187–0.301]*
PlwHIV with medium defences vs. general population	0.474	0.552	0.078	[0.041–0.115]*	0.523	0.622	0.098	[0.058–0.139]*	0.460	0.584	0.124	[0.080–0.167]*
PlwHIV with high defences vs. general population	0.569	0.552	-0.016	[-0.058–0.025]	0.629	0.622	-0.007	[-0.051–0.037]	0.544	0.584	0.040	[-0.00–0.081]
PlwHIV infected through sex vs. general population	0.601	0.552	-0.048	[-0.085- -0.011]*	0.656	0.622	-0.033	[-0.072–0.005]	0.598	0.584	-0.014	[-0.050–0.022]
PlwHIV infected through drug use vs. general population	0.362	0.552	0.190	[0.159–0.220]*	0.388	0.622	0.233	[0.198–0.268]*	0.322	0.584	0.262	[0.220–0.302]*
PlwHIV with a basic education vs. general population with a basic education	0.356	0.436	0.079	[0.049–0.109]*	0.401	0.445	0.043	[0.010–0.077]*	0.345	0.390	0.044	[0.009–0.079]*
PlwHIV with a secondary education vs. general population with a secondary education	0.564	0.544	-0.019	[-0.061–0.023]	0.631	0.609	-0.021	[-0.068–0.025]	0.593	0.557	-0.035	[-0.083–0.011]
PlwHIV with a higher education vs. general population with a higher education	0.761	0.743	-0.017	[-0.081–0.046]	0.848	0.803	-0.044	[-0.113–0.025]	0.763	0.784	0.020	[-0.049–0.091]

Note:

^1^ Employment rate in PlwHIV.

^2^ Employment rate in general population.

^3^ Differences in employment rate between two groups.

*statistically significant at 99%. Source: Designed using data from the Hospital Survey on HIV/AIDS and he Labour Force Survey

In general, PlwHIV characteristics are different from the general population, as they have lower levels of education and there are more males. Therefore, matching techniques allows us to adjust for these differences and obtain robust estimations.

The results obtained from the matching statistical analysis showed that the differences in the employment rate between PlwHIV and the general population with similar sociodemographic characteristics had fallen significantly during the last decade in Spain, especially during the most recent years (during 2009–2010). More precisely, during the period 2001–2002, PlwHIV were less likely to be employed than the general population (Table 3). However, this difference may vary depending on the characteristics of PlwHIV. Overall, PlwHIV were 23.6% less likely to have a job than the general population. This difference was even greater for males, PlwHIV that were males being 33.7% less likely to be employed than the general male population. Likewise, when we compared PlwHIV with a low level of defences, we observed that this group was 31.4% less likely to have a job than the general population (22.5% and 13.0% when the levels of defences were medium and high, respectively). Another significant difference that we discovered was in the case of PlwHIV infected through drug use. For instance, those infected through these means were 34.6% less likely to have a job than the general population (9.0% in the case of those infected through sex). Finally, the education level was also important in explaining the differences in the labour participation of PlwHIV and the general population. More specifically, PlwHIV with a basic education were 39.3% less likely to have a job than their counterparts from the general population (16.7% in the case of middle studies).

**Table 3:** Differences in employment rates between PlwHIV and general population. Results from Propensity Score Matching regressions.

	Period 2001–2002				Period 2006–2007				Period 2009–2010			
Sub-group	Difference	S.E^1^	T-stat	CI 95%	Difference	S.E^1^	T-stat	CI 95%	Difference	S.E^1^	T-stat	CI 95%
PlwHIV vs. general population	-0.236	0.016	-14.59*	-0.204 –-0.267	-0.206	0.018	-11.17*	-0.170 –-0.241	-0.147	0.019	-7.58*	-0,109 –-0.184
PlwHIV female vs. general female population	-0.036	0.032	-1.13	0.026 –-0.098	-0.053	0.037	-1.45	0.019 –-0.125	-0.053	0.037	-1.45	0,019 –-0.125
PlwHIV male vs. general male population	-0.337	0.017	-19.25*	-0.303 –-0.370	-0.261	0.020	-12.76*	-0.221 –-0.300	-0.186	0.022	-8.31*	-0,142 –-0.229
PlwHIV with low defences vs. general population	-0.314	0.029	-10.57*	-0.257 –-0.370	-0.348	0.033	-10.27*	-0.283 –-0.412	-0.288	0.040	-7.04*	-0,209 –-0.366
PlwHIV with medium defences vs. general population	-0.225	0.026	-8.55*	-0.174 –-0.275	-0.185	0.029	-6.19*	-0.128 –-0.241	-0.152	0.032	-4.76*	-0,089 –-0.214
PlwHIV with high defences vs. general population	-0.130	0.029	-4.41*	-0.073 –-0.186	-0.121	0.031	-3.89*	-0.060 –-0.181	-0.048	0.030	-1.60	0,010 –-0.106
PlwHIV infected through sex vs. general population	-0.090	0.025	-3.53*	-0.041 –-0.139	-0.067	0.026	-2.53*	-0.016 –-0.117	-0.025	0.026	-0.95	0,025 –-0.076
PlwHIV infected through drug use vs. general population	-0.346	0.020	-16.49*	-0.306 –-0.385	-0.323	0.025	-12.84*	-0.274 –-0.372	-0.347	0.028	-12.24*	-0,292 –-0.402
PlwHIV with a basic education vs. general population with a basic education	-0.393	0.020	-19.45*	-0.353 –-0.432	-0.420	0.022	-19.07*	-0.376 –-0.463	-0.358	0.024	-14.63*	-0,310 –-0.405
PlwHIV with a secondary education vs. general population with a secondary education	-0.166	0.028	-5.76*	-0.111 –-0.220	-0.182	0.031	-5.87*	-0.121 –-0.242	-0.121	0.033	-3.68*	-0,056 –-0.186
PlwHIV with a higher education vs. general population with a higher education	0.028	0.046	0.61	0.118 –-0.062	0.057	0.049	1.18	0.153 –-0.039	-0.00	0.052	-0.15	0,101 –-0.102

^1^Standard Error. Note: Matching variables used to control: gender, age and level of education.

*statistically significant at 99%. Source: Designed using data from the Hospital Survey on HIV/AIDS and he Labour Force Survey.

During the period 2006–2007, we observed that the differences in the employment rate between PlwHIV and the general population decreased in comparison with those differences in 2001–2002 (Table 2). More precisely, PlwHIV were 20.6% less likely to have a job than the general population. PlwHIV who were males were 26.1% less likely to be employed than the general male population. PlwHIV with a low level of defences were 34.8% less likely to have a job than the general population (18.8% when the levels of defences are medium). Those infected through drugs were 32.3% less likely to have a job than the general population. Finally, PlwHIV with a basic education were 42.0% less likely to have a job than their counterparts from the general population.

**Fig 1:**
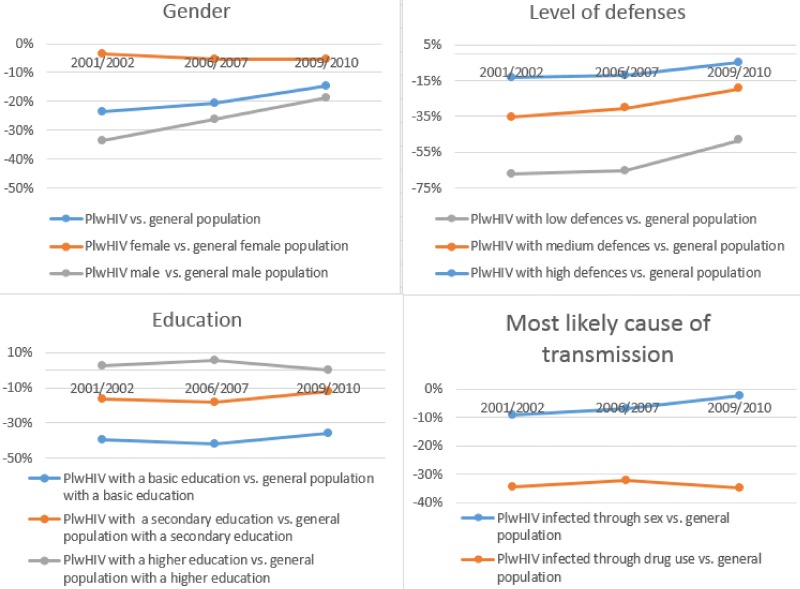
Differences in employment rate between PlwHIV vs general population, by gender, level of defences, education and source of contagious. Results from the matching analysis.

Likewise, during the period 2009–2010, we observed that the differences in the employment rate between PlwHIV and the general population decreased in comparison to those differences in 2001–2002 and 2006–2007 (Table 2, Fig 1). More precisely, PlwHIV were 14.7% less likely to have a job than the general population. PlwHIV who were males were 18% less likely to be employed than the general male population. PlwHIV with a low level of defences was 28.8% less likely to have a job than the general population (15.2% when the levels of defences are medium). Those infected through drugs were 34.7% less likely to have a job than the general population. Finally, PlwHIV with a basic education were 35.8% less likely to have a job than their counterparts from the general population.

In brief, the statistical results obtained showed a general convergence in the employment rate between PlwHIV and general population along business cycle. The employment rate difference evolves from 23.6 percentage point in years 2001–2002 to 14.7 percentage point in 2009–2010. This convergence is mainly explained by two facts. First, the positive evolution of people infected by sex. Thereby, at the end of period, there was not statistical difference in the employment rates between PlwHIV infected through sex and the general population. On contrary, the employment rate of PlwHIV infected through drugs was 34.6 percentage points lower (in 2001–2002) in comparison with general population and 34.7 percentage points (in 2009–2010). Second, the change in the PlwHIV population composition with a decreasing weight of people infected by drug use throughout the decade (from 57.7% in 2001–2002 to 43.2% in 2009–2010). Furthermore, statistical analysis shows that the convergence is significant in those with higher level of defences, males, higher level of education and those infected through sex. In contrast, the differences are still wide among PlwHIV with a low inmunological status of defences, those infected through drugs and those with primary education.

## Discussion

The main contribution that such article can add to the literature is the fact that, firstly, there is no studies in Spain which analyses the difference in the employment rates between PlwHIV and general population along the last business cycle. Secondly, there is no evidence about if the asymmetry in the economic crisis and labour markets across European countries leads to differential results in the convergence of the employment rate between these two different populations. Hence, the aim of this paper was twofold. On the one hand, we aimed to prove whether the employment rates of PlwHIV and general population have converged in Spain along the last business cycle. On the other hand, we wanted to test if the results observed in the two European countries, France and Spain, were similar. The results obtained from the statistical analysis showed, first, that PlwHIV had lower probability of having a job compared with the general population. Second, the differences in the employment rates of PlwHIV and general population has fallen significantly during the last decade in Spain, especially during the most recent years (2009–2010). More precisely, the initial difference in the employment rate between PlwHIV and general population was 23.6 percentage points at the beginning of the period (2001–2002) and 14.7 percentage points at the end (2009–2010). This lower gap in the employment rate between PlwHIV and the general population might be mainly explained by therapeutic advances that have occurred since the 1990s. These advances have substantially increased life expectancy and improved the living standards of this population, bringing their labour situation closer to the general one.

Another relevant conclusion obtained is the fact that the difference in the employment rate between these two populations in all of the periods analysed varies depending on the characteristics of PlwHIV. Concretely, immunological status of defences, source of infection and the level of education play relevant roles in the PlwHIV population when comparing their labour participation with the general population. This finding is in line with the evidence in the literature where it has been proven that the main factors that negatively influence the labour situation among PlwHIV are a poor health status or quality of life, poor job skills experience, low education or being part of vulnerable populations, such as ethnic minorities or intravenous drug users [9].

When we compared our results with those obtained by Annequin et al. [29], we observed that the conclusions are not in line with theirs. While we observed that the differences in employment rate between PlwHIV and general population have decreased during the last decade, they observed that this has increased. Precisely, they concluded that between 2003 and 2011, the employment rate of PlwHIV had declined slightly (60.9% vs. 59.5%), and the unemployment rate had increased (12.6% vs. 15.9%), while the employment and unemployment rates of the general population remained stable (71.9% vs. 71.6%, 5.7% vs. 6.1%, respectively). Part of such differences can be explained by the population included in the analysis as Annequin analysed population older than 25 years old while our study includes people older than 16 years. Another relevant point can be the different composition of French vs. Spanish PlwHIV population and its evolution along the time. Unfortunately, this information is not available in the Annequin et al.’s paper. Furthermore, these differences between the results of evolution of employment rates of the French and Spanish PlwHIV along the business cycle are understandable if we consider that the economic crisis has affected several European countries asymmetrically [43–45], being considerably sharper in effect in the countries of southern Europe and particularly in Spain compared to France. However, the behaviour of labour markets is also highly different. For example, long-term unemployment has traditionally been a source of strong concern in Spain [46]. In the recent economic crisis, unemployment rates have not only grown significantly, as noted, but long-term unemployment has reached a figure never known in Spain: approximately 60% of unemployment has been long-term unemployment [47]. This finding suggests that the rate of unemployment is expected to remain high for many years in Spain, even if there is a strong economic recovery [48]. Some studies [6, 17] have identified that voluntary or compulsory abandonment of employment after diagnosis of HIV can cause a persistent effect in subsequent years. This factor, combined with the strong persistence of long-term unemployment in some countries, can have important economic consequences for PlwHIV and for society. This finding is highly relevant given that as shown in the literature generated in recent years, the precariousness of the work environment and unemployment are factors associated with a worsening of health, especially in the mental dimension [22–27]. Again, an asymmetry in the economic shocks between countries and the different performance of their labour markets will lead to the consequences in that the PlwHIV will differ according to the country and the context analysed. Thus, although our analysis indicates a positive convergence in the employment rate of PlwHIV relative to the general population, employment rates observed at the end of our study period were lower than those observed at the end of the cycle of economic growth (years 2006–2007) and, in any case, they are lower than those observed by Annequin et al [29] for France in the same years, as the employment rate of PlwHIV in France reached 60% during 2009–2010, while in Spain, it was barely 47%.

Several limitations of our study should be discussed. First, we used cross-sectional surveys that were conducted over a decade, in which the last year available is 2010. In this sense, it would have been useful to conduct a longitudinal survey so that we could control some of the individuals’ unobservable heterogeneity and to check the evolution of the employment rate in subsequent years of economic crisis after 2010. A future line of research would be to prove whether the trend found in this study until 2010 would remain through the 2011–2016 years. Nevertheless, we wish to highlight that the Hospital Survey on HIV/AIDS provides valuable information, as it can control the effects of the observable elements that are the sources of such heterogeneity (age, gender, education level, most likely cause of transmission, and immunological status of defences). Another issue that should be mentioned is that we employ the term labour participation as synonymous of having a job. Ideally, it would have been preferable to distinguish between three different states: employed, unemployed and inactive as Annequin et al [29] done. Unfortunately, the Hospital Survey on HIV/AIDS does not raise questions about labour participation in the same way as the Labour Force Surveys in Spain and several European countries do. Thus, the description of an unemployed person, as defined in the LFS (unemployed person actively seeking and willing to immediately accept a job) does not reflect the description in the Hospital Survey on HIV/AIDS (where the answer is selected by an individual from a set of occupational categories). Moreover, information related to wages and non-labour income was not available. Wages in the labour market are a relevant variable to be considered when analysing the labour supply. However, in the case of PlwHIV, it should be noted that evidence of the effect of wages on PlwHIV labour participation is scarcer and non-conclusive [11, 49, 50]. Finally, another limitation is related to the fact that it was not possible to get some relevant information that might be quite relevant when calculating the propensity scores. For instance, we could not get information on geography, race/ethnicity or immigrant status”. Such socio-economic outcomes may be affected matches.

Despite these limitations, this study provides both relevant information on the employment status of PlwHIV in recent years and an appropriate direction for public policies. Even though it has experienced a significant convergence in the employment rate between PlwHIV and the general population along the business cycle, there is still a relevant gap in the labour market of this target population. More precisely, the obtained figures highlight the significant differences detected in PlwHIV and the general population based mainly on their individual characteristics, demonstrating that optimal policy design should integrate both healthcare policies (early diagnosis, access to treatment, psychological support) and other transversal measures focused mainly on workplace and education in order to support PlwHIV and improve their job opportunities. In this context, it is necessary to improve the existing databases of PlwHIV performing longitudinal surveys that include more precise labour supply information (wages, employment status of other family members, job sector) and consider the context of business cycle and institutional characteristics of labour markets of each country.
